# External ventricular drain use is associated with functional outcome in aneurysmal subarachnoid hemorrhage

**DOI:** 10.1186/s42466-022-00189-6

**Published:** 2022-06-27

**Authors:** Sarah E. Nelson, Jose I. Suarez, Alexander Sigmon, Jun Hua, Casey Weiner, Haris I. Sair, Robert D. Stevens

**Affiliations:** 1 Departments of Neurosurgery and Neurology, Mount Sinai Health System, 1000 10th Avenue, New York, NY 10019 USA; 2 Departments of Neurology, Neurosurgery, and Anesthesiology and Critical Care Medicine, Johns Hopkins University, Baltimore, USA; 3 Department of Neurosciences, Johns Hopkins University, Baltimore, USA; 4 Department of Radiology and Radiological Science, Johns Hopkins University, Baltimore, USA; 5 F.M. Kirby Research Center for Functional Brain Imaging, Kennedy Krieger Institute, Johns Hopkins University, Baltimore, USA; 6 Department of Biomedical Engineering, Johns Hopkins University, Baltimore, USA

**Keywords:** Ventriculostomy, Aneurysmal subarachnoid hemorrhage, Sex difference, Outcome assessment

## Abstract

**Purpose:**

External ventricular drains (EVD) are commonly used in aneurysmal subarachnoid hemorrhage (aSAH) patients and can be life-saving by diverting cerebrospinal fluid. However, the overall relationship between EVD use and outcome is poorly understood.

**Methods:**

In an exploratory analysis of an aSAH patient cohort, we examined EVD use in relation to modified Rankin Scale (mRS) at hospital discharge and at 6 months (unfavorable outcome = mRS > 2) using univariable and multivariable analyses.

**Results:**

EVDs were placed in 31 of 56 (55.4%) patients and more often in women than men (66.7% vs 35.0%, *p* = 0.022) despite similar rates of hydrocephalus. Women had greater ICU [18 (13.5–25) vs 11.5 (6.5–18.5) days, *p* = 0.014] and hospital lengths of stay (LOS) [20.5 (16.5–34) vs 13.5 (10.5–27) days, *p* = 0.015] than men and greater mRS at discharge [4 (3–5) vs 3 (2–3.5), *p* = 0.011] although mRS at 6 months was similar. Patients with EVDs had longer ICU and hospital LOS and greater mRS at discharge [5 (3–6) vs 2 (2–3), *p* < 0.001] and at 6 months [4 (2–6) vs 1 (0–2), *p* = 0.001] than those without an EVD. In multivariable models, EVD use was associated with unfavorable 6-month outcome accounting for age, sex, and admission modified Fisher scale, but not in models adjusting for Hunt and Hess scale and World Federation of Neurological Surgeons scale.

**Conclusion:**

In an aSAH cohort, the use of EVDs was associated with female sex and longer LOS, and may be linked to functional outcomes at discharge and at 6 months, although these associations warrant further investigation.

## Introduction

Despite advances in management, aneurysmal subarachnoid hemorrhage (aSAH) continues to be associated with high morbidity and mortality [1]. A substantial proportion (recent case series suggest 22–74% [2, 3]) of aSAH patients undergo cerebrospinal fluid diversion via an external ventricular drain (EVD). An EVD can be lifesaving—for example in the management of hydrocephalus and elevated intracranial pressure [3]. However, EVDs are associated with a number of complications including intracranial hemorrhage, syndromes of overdrainage and underdrainage, and infection [4, 5]. The overall association between EVD use and outcome following aSAH is complex and not well understood [3]. Here, we explored the relationship between EVD use and outcomes in a cohort of patients with aSAH. This exploratory analysis suggests hypotheses to be tested in future studies evaluating the use of EVDs in aSAH patients, including EVD duration and the need for ventriculoperitoneal shunt (VPS).

## Materials and methods

### Population

We retrospectively analyzed data from a prospective observational cohort study of aSAH patients admitted between 2017 and 2020 to the Neuroscience Critical Care Units at The Johns Hopkins Hospital and at Johns Hopkins Bayview Medical Center. This study enrolled adult patients with aSAH of all clinical grades who underwent brain MRI and longitudinal follow-up, and in whom no contraindication to MRI was found. The MRI database was created with approval from the Johns Hopkins Medicine Institutional Review Board under two different protocols, one of which had waived the need for consent; hence not all patients in the study required written informed consent. Independent results on some of these patients were reported in a recent paper by our group [6]. Variables extracted were demographic (e.g., age, sex) and clinical data (e.g., admission Glasgow Coma Scale (GCS), Hunt and Hess scale (HH), modified Fisher scale (mF), World Federation of Neurological Surgeons scale (WFNS)), occurrence of hydrocephalus (defined if increased ventricular size or hydrocephalus was mentioned in the radiology report of any neuroimage at any time during the patient’s hospitalization), aneurysm location and type of securement, placement of an EVD, occurrence of delayed cerebral ischemia (DCI) or vasospasm (if either diagnosis was discussed in the electronic medical record), occurrence of ventriculitis, occurrence of EVD tract hemorrhage (defined as described before [7]), occurrence of overdrainage (as evidenced by extra-axial fluid collections felt likely due to EVD drainage), and placement of a VPS. Reason for EVD insertion was abstracted from the electronic medical record. ICU and hospital lengths of stay (LOS) were calculated for each patient. Modified Rankin Scale (mRS) at discharge was abstracted from the chart. Six months after discharge, SEN contacted patients by telephone to assess their mRS as has been done previously [8, 9]; in some cases, mRS was abstracted from the chart if unable to be obtained over the phone. Notably, prior to EVD insertion, our center routinely evaluates, and corrects, for the presence of a coagulopathy or antithrombotic use (e.g., by administering reversal agents for anticoagulants). Also, we utilize a gradual EVD weaning strategy that occurs over days. All aSAH patients had access to the same rehabilitation resources after hospitalization, although their needs differed depending on their discharge functional status. The data abstractors (SEN, AS) were not blinded to patient treatment and to outcome data.

### Statistical analysis

The sample size was one of convenience and was determined by patients available in the aSAH MRI cohort database; no sample size or power analysis was computed. The Shapiro–Wilk test revealed that all continuous data were nonparametric (*p* < 0.05) except age, admission mF, and admission HH. Therefore, Students t-test (for age, admission mF, and admission HH) and Wilcoxon rank-sum tests (for nonparametric data) were performed for continuous variables; chi-square tests were used for non-continuous data. Characteristics of men and women in the dataset were compared as well as those who received an EVD and those who did not.

The two principal outcomes were discharge and 6-month functional status assessed using a dichotomized mRS with favorable outcome defined as a mRS ≤ 2; secondary outcomes were ICU and hospital LOS. Multivariable logistic regression analyses were performed evaluating age (since it can contribute to outcome in aSAH [10]), sex, presence of EVD (the latter two because of their focus in this study), hydrocephalus, delayed cerebral ischemia/vasospasm (the latter two because they were significant in univariate analysis between those with EVDs and those without), occurrence of EVD tract hemorrhage (which was statistically significantly different between men and women), overall EVD complications (defined here as ventriculitis + tract hemorrhage + overdrainage) and validated admission scales (mF, HH, GCS, or WFNS). Those variables tested showed no evidence for interaction (sex*EVD, EVD*hospital length-of-stay, EVD*ICU length-of-stay, and EVD*hydrocephalus). Model goodness-of-fit was assessed using the Hosmer–Lemeshow test. Missing data were not imputed. Data were analyzed in Stata 15.1 (College Station, TX, USA). Statistical significance was set at *p* < 0.05.

## Results

A total of 56 patients were enrolled of whom 31 (55.4%) received an EVD. Median (IQR) duration of EVD was 18.9 (13–22) days, and 6-month follow-up was obtained in all but 6 patients in the total dataset (due either to being unable to reach the patient and/or no follow-up notes in the electronic medical record). The most common reasons for EVD placement were hydrocephalus (n = 29), altered mental status (n = 28), and presence of intraventricular hemorrhage (n = 6) (patients’ charts often indicated more than one reason for EVD placement). Mechanical ventilation occurred for 9 (3.5–17.5) days in those who had EVDs placed. In all patients with an EVD, mean and median intracranial pressures readings, obtained for this study only on the date of MRI, were < 20 mmHg.

Women and men had similar admission mF, HH, GCS, and WFNS on admission (Table 1). Despite similar rates of hydrocephalus, women were more likely to receive an EVD (66.7% vs 35.0%, *p* = 0.022). Women had longer ICU LOS [18 (13.5–25) vs 11.5 (6.5–18.5) days, *p* = 0.014] and hospital LOS [20.5 (16.5–34) vs 13.5 (10.5–27) days, *p* = 0.015] as well as higher mRS at discharge [4 (3–5) vs 3 (2–3.5), *p* = 0.011], although mRS at 6 months was similar between both sexes. Among patients who had EVDs placed, there was no difference between sexes in rates of ventriculitis or VPS placement but women were more likely to suffer from EVD tract hemorrhage (75.0% vs 14.3%, *p* = 0.004) and overall EVD complications (ventriculitis plus tract hemorrhage plus overdrainage; 79.2% vs 14.3%; *p* = 0.002). Duration with EVD was similar between sexes [18 (13.5–24) vs 14 (10–22) days, *p* = 0.24].

**Table 1:** Differences between men and women

	Men (n = 20)	Women (n = 36)	*P* value
Age^a^	55.9 ± 16.4	59.1 ± 10.5	0.86
Admission mF^a^	2.7 ± 1.1	3.3 ± 1.1	0.056
Admission HH^a^	2.5 ± 1.7	2.9 ± 1.3	0.33
Admission GCS	14.5 (6.5–15)	13 (8.5–15)	0.35
Admission WFNS	1.5 (1–4.5)	2 (1–4)	0.45
Hydrocephalus	11 (55.0%)	28 (77.8%)	0.076
EVD+	7 (35.0%)	24 (66.7%)	0.022
Aneurysm treatment			0.082
Clip	2 (10.0%)	12 (33.3%)	
Coil	9 (45.0%)	16 (44.4%)	
Other^b^	9 (45.0%)	8 (22.2%)	
Days from SAH to MRI	5.5 (3.5–11.5)	7 (3.5–13.5)	0.64
DCI or vasospasm	8 (40.0%)	21 (58.3%)	0.19
ICU LOS	11.5 (6.5–18.5)	18 (13.5–25)	0.014
Hospital LOS	13.5 (10.5–27)	20.5 (16.5–34)	0.015
Days with EVD among EVD+	14 (10–22)	18 (13.5–24)	0.24
EVD complications	1 (14.3%)	19 (79.2%)	0.002
Ventriculitis	0 (0%)	3 (12.5%)	0.33
Tract hemorrhage	1 (14.3%)	18 (75.0%)	0.004
Overdrainage	0 (0%)	3 (12.5%)	0.33
Received VPS among EVD+	2 (28.5%)	6 (25.0%)	0.85
mRS at discharge	3 (2–3.5)	4 (3–5)	0.011
mRS at 6 months	1.5 (0–5)	2 (1–5)	0.65

Data presented are either n (%) or median (IQR) except where noted otherwise

mF, Modified Fisher Scale; HH, Hunt and Hess Scale; GCS, Glasgow Coma Scale; WFNS, World Federation of Neurological Surgeons Scale; EVD+, received external ventricular drain; SAH, subarachnoid hemorrhage; MRI, magnetic resonance imaging; DCI, delayed cerebral ischemia; ICU, intensive care unit; LOS, length of stay; mRS, modified Rankin Scale

^a^Mean ± standard deviation

^b^Other refers to pipeline (n = 1), glue embolization (n = 1), both clipping and coiling (n = 1), or neither (n = 14)

Patients who received an EVD had greater mF, HH, and WFNS as well as worse GCS on admission (all *p* < 0.001) and were more likely to be diagnosed with hydrocephalus (*p* < 0.001) than those who did not receive an EVD (Table 2). Those with an EVD were more likely to suffer from DCI or vasospasm (*p* = 0.001). In addition, their ICU LOS [22 (18–30) vs 10 (8–14) days, *p* < 0.001] and hospital LOS [30 (19–46) vs 14 (12–17) days, *p* < 0.001] as well as mRS at discharge [5 (3–6) vs 2 (2–3), *p* < 0.001] and at 6 months [4 (2–6) vs 1 (0–2), *p* = 0.001] were greater than those who did not receive an EVD.

**Table 2:** Differences between patients who received EVDs and those who did not

	EVD+ (n = 31)	EVD− (n = 25)	*P* value
Age^a^	59.0 ± 13.4	56.7 ± 12.5	0.60
Female sex	24 (77.4%)	12 (48.0%)	0.022
Admission mF^a^	3.6 ± 0.7	2.3 ± 1.1	< 0.001
Admission HH^a^	3.5 ± 1.3	1.8 ± 0.9	< 0.001
Admission GCS	9 (5–14)	15 (14–15)	< 0.001
Admission WFNS	4 (2–5)	1 (1–2)	< 0.001
Hydrocephalus	31 (100.0%)	8 (32.0%)	< 0.001
Aneurysm treatment			0.88
Clip	7 (22.6%)	7 (28.0%)	
Coil	14 (45.2%)	11 (44.0%)	
Other^b^	10 (32.3%)	7 (28.0%)	
Days from SAH to MRI	8 (4–16)	6 (3–11)	0.23
DCI or vasospasm	22 (71.0%)	7 (28.0%)	0.001
ICU LOS	22 (18–30)	10 (8–14)	< 0.001
Hospital LOS	30 (19–46)	14 (12–17)	< 0.001
EVD complications	20 (64.5%)	N/A	N/A
Ventriculitis	3 (9.7%)	N/A	N/A
Tract hemorrhage	19 (61.3%)	N/A	N/A
Overdrainage	3 (9.7%)	N/A	N/A
Received VPS among EVD+	8 (25.8%)	N/A	N/A
mRS at discharge	5 (3–6)	2 (2–3)	< 0.001
mRS at 6 months	4 (2–6)	1 (0–2)	0.001

Data presented are either n (%) or median (IQR) except where noted otherwise.

mF, Modified Fisher Scale; HH, Hunt and Hess Scale; GCS, Glasgow Coma Scale; WFNS, World Federation of Neurological Surgeons Scale; EVD+, received external ventricular drain; EVD-, did not receive external ventricular drain; SAH, subarachnoid hemorrhage; MRI, magnetic resonance imaging; DCI, delayed cerebral ischemia; ICU, intensive care unit; LOS, length of stay; mRS, Modified Rankin Scale; N/A, not applicable

^a^mean ± standard deviation

^b^Other refers to pipeline (n = 1), glue embolization (n = 1), both clipping and coiling (n = 1), or neither (n = 14)

In multivariable logistic regression models for unfavorable outcome at discharge, sex was an independent predictor (*p* = 0.049) when adjusting for age and admission WFNS but not in models that adjusted for age, admission mF, GCS, or HH (Table 3). It should be noted that admission mF, HH, and WFNS were also predictors of discharge outcome in models containing sex or both age and sex. In multivariable models evaluating EVD use, presence of EVD was the only independent predictor of unfavorable outcome at discharge in models that also contained either admission mF (*p* = 0.008) or admission HH (*p* = 0.041). Neither sex nor EVD use were independent predictors of discharge functional outcome in models that either contained ICU or hospital LOS in addition to admission mF, GCS, HH, or WFNS. Notably, EVD use remained an independent predictor of unfavorable outcome at hospital discharge when presence of hydrocephalus was included in models with age and sex, although this was no longer the case when clinical scores were included in the models (GCS, HH, WFNS).

**Table 3:** Odds ratios in multivariable models for the outcome mRS > 2 at discharge

Variable	Model including admission mF		Model including admission GCS		Model including admission HH		Model including admission WFNS	
	OR	95% CI	OR	95% CI	OR	95% CI	OR	95% CI
Model 1								
Age	1.07	0.99–1.16	1.05	0.97–1.13	1.07	0.99–1.15	1.04	0.97–1.13
Sex	2.97	0.59–14.81	2.60	0.50–13.4	2.13	0.39–11.56	3.01	0.55–16.32
Admission mF	0.92	0.42–2.01	NI	NI	NI	NI	NI	NI
Admission GCS	NI	NI	0.63	0.28–1.45	NI	NI	NI	NI
Admission HH	NI	NI	NI	NI	1.93	0.73–5.07	NI	NI
Admission WFNS	NI	NI	NI	NI	NI	NI	2.56	0.74–8.86
EVD	45.37	2.69–765.69	11.12	0.71–174.82	15.33	1.11–210.92	9.31	0.59–145.74
Model 2								
Age	1.09	0.99–1.19	1.07	0.98–1.17	1.09	0.99–1.20	1.07	0.97–1.17
Sex	2.94	0.53–16.10	2.96	0.49–17.88	2.18	0.38–12.53	3.10	0.51–18.94
Admission mF	0.81	0.34–1.93	NI	NI	NI	NI	NI	NI
Admission GCS	NI	NI	0.62	0.31–1.25	NI	NI	NI	NI
Admission HH	NI	NI	NI	NI	1.87	0.76–4.59	NI	NI
Admission WFNS	NI	NI	NI	NI	NI	NI	2.56	0.79–8.30
EVD	17.17	0.93–317.69	1.38	0.04–44.87	4.01	0.20–79.01	1.71	0.05–55.51
ICU LOS	1.11	0.96–1.27	1.14	0.96–1.34	1.11	0.95–1.30	1.12	0.95–1.32
Model 3								
Age	1.09	0.99–1.21	1.07	0.97–1.19	1.09	0.98–1.21	1.07	0.97–1.18
Sex	3.13	0.50–19.40	3.41	0.48–24.18	2.37	0.36–15.72	3.25	0.47–22.31
Admission mF	0.80	0.32–1.97	NI	NI	NI	NI	NI	NI
Admission GCS	NI	NI	0.61	0.30–1.25	NI	NI	NI	NI
Admission HH	NI	NI	NI	NI	1.91	0.74–4.89	NI	NI
Admission WFNS	NI	NI	NI	NI	NI	NI	2.51	0.70–8.97
EVD	14.68	0.99–217.27	1.24	0.05–31.41	3.67	0.26–51.39	1.57	0.06–44.22
Hospital LOS	1.11	0.98–1.25	1.13	0.98–1.31	1.12	0.98–1.28	1.12	0.97–1.29

Model 1: age, sex, EVD. Model 2: age, sex, EVD, ICU LOS. Model 3: age, sex, EVD, hospital LOS

OR, odds ratio; CI, confidence interval; mF, Modified Fisher Scale; HH, Hunt and Hess Scale; GCS, Glasgow Coma Scale; WFNS, World Federation of Neurological Surgeons Scale; EVD, external ventricular drain; ICU, intensive care unit; LOS, length of stay; mRS, Modified Rankin Scale

When evaluating unfavorable outcome at 6 months, sex was not an independent predictor while admission scores were (all *p* < 0.05) (Table 4). EVD use remained an independent predictor of outcome in models that adjusted for age, sex, and admission mF (*p* = 0.027) but not in models that included admission GCS, HH, or WFNS. When adjusting for EVD use and ICU or hospital LOS in multivariable models, admission HH, GCS, and WFNS remained independent predictors of unfavorable outcome at 6 months (all *p* < 0.05), presence of EVD was not a predictor, and hospital LOS emerged as an independent predictor in models that included age, sex, EVD use, and admission GCS (*p* = 0.032) or WFNS (*p* = 0.044). EVD use was not a predictor of unfavorable outcome at 6 months in models that contained hydrocephalus in addition to age, sex, and EVD use; further, clinical scores (GCS, HH, WFNS) were independent predictors when they were also included in multivariable models. Multivariable models containing delayed cerebral ischemia/vasospasm demonstrated that this variable was not an independent predictor for unfavorable outcome either at discharge or at 6 months.

**Table 4:** Odds ratios in multivariable models for the outcome mRS > 2 at 6 months

Variable	Model including admission mF		Model including admission GCS		Model including admission HH		Model including admission WFNS	
	OR	95% CI	OR	95% CI	OR	95% CI	OR	95% CI
Model 1								
Age	1.03	0.97–1.08	1.01	0.94–1.07	1.02	0.96–1.08	1.00	0.94–1.07
Sex	0.49	0.10–2.56	0.96	0.15–6.25	0.65	0.10–4.17	0.87	0.14–5.40
Admission mF	1.29	0.56–2.93	NI	NI	NI	NI	NI	NI
Admission GCS	NI	NI	0.73	0.56–0.95	NI	NI	NI	NI
Admission HH	NI	NI	NI	NI	2.41	1.22–4.74	NI	NI
Admission WFNS	NI	NI	NI	NI	NI	NI	2.39	1.24–4.60
EVD	8.32	1.27–54.27	2.29	0.32–16.61	2.89	0.46–18.21	1.97	0.25–15.34
Model 2								
Age	1.04	0.98–1.10	1.02	0.94–1.09	1.03	0.97–1.11	1.01	0.94–1.08
Sex	0.40	0.07–2.15	0.85	0.13–5.57	0.51	0.08–3.25	0.79	0.13–4.97
Admission mF	1.34	0.57–3.14	NI	NI	NI	NI	NI	NI
Admission GCS	NI	NI	0.66	0.49–0.90	NI	NI	NI	NI
Admission HH	NI	NI	NI	NI	2.64	1.27–5.51	NI	NI
Admission WFNS	NI	NI	NI	NI	NI	NI	3.00	1.39–6.47
EVD	4.47	0.54–37.00	0.45	0.03–6.91	1.07	0.10–11.21	0.45	0.03–6.60
ICU LOS	1.04	0.97–1.13	1.09	0.98–1.20	1.06	0.97–1.17	1.08	0.98–1.18
Model 3								
Age	1.06	0.99–1.14	1.03	0.95–1.12	1.05	0.97–1.14	1.03	0.95–1.11
Sex	0.41	0.07–2.30	1.02	0.15–7.03	0.56	0.09–3.46	0.86	0.13–5.83
Admission mF	1.26	0.51–3.07	NI	NI	NI	NI	NI	NI
Admission GCS	NI	NI	0.66	0.47–0.92	NI	NI	NI	NI
Admission HH	NI	NI	NI	NI	2.61	1.21–5.61	NI	NI
Admission WFNS	NI	NI	NI	NI	NI	NI	2.83	1.29–6.18
EVD	3.59	0.45–28.58	0.44	0.03–5.75	0.90	0.10–8.38	0.52	0.04–6.48
Hospital LOS	1.06	1.00–1.12	1.08	1.01–1.16	1.07	1.00–1.15	1.07	1.00–1.15

Model 1: age, sex, EVD. Model 2: age, sex, EVD, ICU LOS. Model 3: age, sex, EVD, hospital LOS

OR, odds ratio; CI, confidence interval; mF, Modified Fisher Scale; HH, Hunt and Hess Scale; GCS, Glasgow Coma Scale; WFNS, World Federation of Neurological Surgeons Scale; EVD, external ventricular drain; ICU, intensive care unit; LOS, length of stay; mRS, Modified Rankin Scale

Including EVD tract hemorrhage or overall EVD complications in multivariable models instead of presence of EVD (to avoid collinearity) revealed either inability of the models to run due to small sample size (in the case of unfavorable outcome at discharge) or no independent predictors of unfavorable outcome (in the case of 6-month unfavorable outcome). The Hosmer–Lemeshow test for goodness-of-fit revealed that the completed multivariable logistic regression models fit reasonably well (all *p* > 0.05).

## Discussion

In this study, EVD use was associated with higher ICU and hospital LOS and potentially worse functional outcome at discharge and at 6 months, although the latter relationship did not hold in models adjusting for measures of clinical severity. EVDs were more common in women despite similar rates of hydrocephalus, and women had greater ICU and hospital LOS as well as worse functional outcome at discharge. We found that sex may be an independent predictor of discharge outcome. In models that adjusted for hydrocephalus, EVD use remained an independent predictor of functional outcome at discharge but not at 6 months.

EVDs are frequently placed in patients with aSAH, yet optimal EVD management is a subject of debate, and currently there are no definitive guidelines. Studies have shown that aSAH patients in whom EVDs are placed seem to improve clinically in the short-term; however, the association between EVD use and long-term outcomes remains unclear [3, 11–13]. EVD placement in SAH patients seems to be associated with improved long-term outcomes in some studies, [13, 14] and presence of hydrocephalus was associated with decreased hospital mortality in another study (likely due to prompt treatment with EVDs) [15]. However, a study by Gerner et al. demonstrated that a greater proportion of non-survivors had EVDs than survivors, and that need for VPS was associated with decreased likelihood of favorable functional outcome, reduced chance of return to work, and decreased self-reported health [2]. Further, EVDs are associated with considerable risk as evidenced by the occurrence of ventriculitis, the incidence of which increases substantially the longer an EVD remains in place [4]). There appears to be high variability in EVD discontinuation practices in aSAH patients [16]. For all conditions requiring an EVD, the Neurocritical Care Society recommends that an EVD be weaned as quickly as possible to minimize the risk of infection, although they acknowledge that rapid and gradual EVD weaning strategies may lead to similar outcomes [4]. Regarding EVD management in aSAH patients specifically, a randomized study showed similar rates for VPS placement for aSAH patients in both gradual versus rapid weaning strategy groups, and further that ICU and hospital days were longer for those in the gradual weaning group [17]. The American Heart Association has stated that, for aSAH patients, weaning an EVD over 24 h does not seem to reduce the need for cerebrospinal fluid shunting [10]. A multi-institutional survey suggested that most institutions favored a gradual weaning approach for EVDs in aSAH [18]. Overall, it seems that early EVD clamping can lead to a shorter length of stay and fewer complications [19]. A recent study showed an association between EVD use and various outcomes; in particular a rapid EVD wean (as compared to gradual) was associated with decreased ICU length of stay and rates of VPS placement [20].

In this preliminary study, women were more likely to receive EVDs despite similar rates of hydrocephalus and admission examinations. We also found that women more frequently had EVD tract hemorrhage than men. Women are known to have a higher incidence of aSAH than men, and studies have suggested that women may have higher mortality following aSAH [10]. However, a retrospective analysis of over 600 aSAH patients found no difference between men and women in either admission clinical examination as defined by WFNS or in outcome as defined by mRS and LOS [21]. In this same study, women and men had similar rates of hydrocephalus, though EVD use by sex was not reported [21]. The significance of the greater instance of EVD tract hemorrhage in women in our cohort is unclear; prior studies that statistically compared this complication between men and women have not found this difference [22–24].

Our data further suggest that despite relatively low rates of ventriculoperitoneal shunting, EVD use may delay patient discharge from the hospital and may be associated with worse functional status at time of discharge and at 6 months. These associations with outcome did not seem to be mediated by EVD complications, and the association with discharge outcome remained even in models that included hydrocephalus. Of note, however, the link between EVD use and 6-month outcome was not significant in most models that adjusted for clinical admission scales, which remain the best predictors of outcome.

Specific limitations of this study include its single center design, small sample size, and the analysis of patients who were enrolled in a separate prospective cohort study with specific inclusion criteria, which may limit inference and generalizability. Data abstractors were not blinded to study variables, which could bias our results. In addition, SEN has undergone periodic mRS training throughout her career and was thus familiar with this approach, although we have realized that her mRS certification was not current at the time she collected mRS data for this study. Furthermore, we did not collect systemic data (e.g., cardiac and pulmonary complications) that can also impact prognosis in aSAH. Nonetheless, a strength of our study is the large number of variables regarding patients that permitted statistical analyses including multivariable logistic models. Even in this moderate number of aSAH patients, our results suggest that EVD use may harm patients in the short-run suggesting a potentially actionable item for clinicians who treat aSAH patients.

## Conclusions

In this study, EVD use was associated with higher ICU and hospital LOS as well as greater mRS at discharge and at 6 months; nonetheless, clinical severity scores proved to be better predictors of mRS at 6 months in multivariable models. EVDs were more frequently placed in women than men despite similar rates of hydrocephalus, and women had greater ICU and hospital LOS as well as higher mRS at discharge. Our overall results should be interpreted with caution as this was an exploratory analysis and deserves further evaluation in larger studies.

## Data Availability

Available from corresponding author upon reasonable request.
